# Species Identification of Food Contaminating Beetles by Recognizing Patterns in Microscopic Images of Elytra Fragments

**DOI:** 10.1371/journal.pone.0157940

**Published:** 2016-06-24

**Authors:** Su Inn Park, Halil Bisgin, Hongjian Ding, Howard G. Semey, Darryl A. Langley, Weida Tong, Joshua Xu

**Affiliations:** 1 Department of Computer Science, Texas A&M University, College Station, Texas, United States of America; 2 Division of Bioinformatics and Biostatistics, National Center for Toxicological Research, U.S. Food and Drug Administration, Jefferson, Arkansas, United States of America; 3 Arkansas Regional Laboratories, Office for Regulatory Affairs, U.S. Food and Drug Administration, Jefferson, Arkansas, United States of America; National Chiao Tung University, TAIWAN

## Abstract

A crucial step of food contamination inspection is identifying the species of beetle fragments found in the sample, since the presence of some storage beetles is a good indicator of insanitation or potential food safety hazards. The current pratice, visual examination by human analysts, is time consuming and requires several years of experience. Here we developed a species identification algorithm which utilizes images of microscopic elytra fragments. The elytra, or hardened forewings, occupy a large portion of the body, and contain distinctive patterns. In addition, elytra fragments are more commonly recovered from processed food products than other body parts due to their hardness. As a preliminary effort, we chose 15 storage product beetle species frequently detected in food inspection. The elytra were then separated from the specimens and imaged under a microscope. Both global and local characteristics were quantified and used as feature inputs to artificial neural networks for species classification. With leave-one-out cross validation, we achieved overall accuracy of 80% through the proposed global and local features, which indicates that our proposed features could differentiate these species. Through examining the overall and per species accuracies, we further demonstrated that the local features are better suited than the global features for species identification. Future work will include robust testing with more beetle species and algorithm refinement for a higher accuracy.

## Introduction

In the food inspection process, insects are a crucial indicator of food sanitation, contamination, and other potential food safety problems. The insect species identification process involves time-consuming microscopic comparison of the insect fragments recovered from the food sample with reference insect specimens. Accurate identification of insects is important for regulatory purposes in order to evaluate the etiology of the food contamination as well as the degree of potential risk to the consumer [1]. There are about 600 insect species that attack food, the majority of which belong to the order Coleoptera (beetles) [2, 3]. Since some insect species, particularly those belonging to the same genus, are very similar in appearance, species identification of food contaminating beetles remains a major challenge.

Current attempts to recover insect fragments and identify their species rely on various micro-analytical techniques, such as chemical extraction or flotation methods [4]. Based on our internal lab experience, about 80% of the fragments recovered were noted to be from storage beetles, out of which about one quarter were reported to be the hardened forewings (elytra). The elytra are of special interest as they contain more recognizable patterns than other exoskeleton fragments. Due to the high degree of similarity among the beetles, many years of experience are required for food inspection analysts to master the microscopic details sufficiently. Even taking into account this consideration, the identification results may vary depending on the analyst’s level of expertise. Compounding this difficulty is the fact that the recovered elytra fragments are usually broken, with some damage to the morphological characteristics. In the face of such complications, the task of species identification through fragments is very challenging; it is critical to develop an efficient, consistent, and reliable approach.

Our innovative solution is to take advantage of modern technologies to build a computer system to identify the species of a beetle by recognizing patterns on the elytra fragments, which are frequently detected during the food inspection process. Thus, we are taking an interdisciplinary approach consisting of image processing [5], machine learning [6], and entomology to address this food safety challenge.

As a pilot effort, we collected whole elytra specimens of 15 most common storage beetle species and their sample microscopic images; 3 to 6 specimens per species were then chosen along with their images. Next, we simulated elytra fragments by randomly excerpting subimages (i.e., with random position, width, and height) from the sample images; pattern features were subsequently extracted from each subimage. After extraction, the resulting features were utilized as inputs to train and test artificial neural networks (ANNs) to assign the class label for a given specimen. The rest of the paper is organized as follows: we describe the existing studies and systems designed for insect/fly recognition in Section 2; we explain the steps for preprocessing and feature extraction [7] in detail and demonstrate how ANNs are constructed with the feature sets in Section 3; we report our experimental results in Section 4; finally, we discuss the performance and findings of the proposed framework, draw conclusions, and propose some future directions in Section 5.

## Related Work

Although the identification of insects can be successfully achieved through chemical or genetic procedures, computer-aided approaches have been seen as faster and more cost effective. Therefore, image analysis has been used in various studies that focused on entomology research. One of the early examples is an image-based identification system proposed by Weeks et al.; using this system, they were able to discriminate five closely related species of Ichneumonidae [8]. They extracted the principal components of the wing images and measured the similarities by comparing the characteristics of these components. Their model achieved 94% accuracy for 175 wings.

In another work [9], bee species were identified by examining forewing features and wing venation. The authors constructed features starting from the lines and intersections in the image; after refining the feature set, they applied support vector machine (SVM) and kernel discriminant analysis (KDA) methods for classification. The distinctive nature of wing patterns also inspired a classification method for fruit flies such as Rhagoletis pomonella, which has four sibling species [10]. The authors hypothesized that hidden biological information existed in the vein structure of wings, which in turn could make the fly distinguishable. Therefore, they utilized Bayesian and probability neural networks, adjusted for the size and shape of the wing.

An automated taxonomic identification of stoneflies was studied by Larios et al., who employed a four-step process consisting of a region of interest, representation of these regions as scale-invariant feature transform (SIFT) vectors [11], classification of SIFT vectors into features to form a histogram of detected features, and classification of the feature histograms [12]. Furthermore, the authors determined the principal curvature-based region (PCBR) for the first step; they were able to discriminate four classes at 82% accuracy. When they considered two closely-related taxa, Calineuria and Doroneuria, as one class, they reported a higher accuracy of 95%. Wang et al. presented a system which was primarily designed for identification of insects at the order level. They defined several features and used artificial neural networks (ANNs) and an SVM model to analyze these features for identification [13].

Besides the abovementioned techniques, semi-automated digital library systems have been also introduced. However, most of these systems require the user’s domain knowledge and control to complete the task. For instance, Caci et al. utilizes the I3S software package [14], which allows the user to compare an unknown image with images in a reference library using the contours [15]. The authors used this system to identify the beetle Rosalia alpina for which they had 290 samples, each sample having 2 images. Another stand-alone tool, digital automated identification system (DAISY), requires the user to capture the image and perform segmentation [16], due to the need for identical image alignment. This requirement comes from DAISY’s visual recognition approach, which relies on orthogonal eigenimages (principal components) [17]. DAISY was further used for the identification of live moths [18].

As a summary, prior research or systems on beetle/insect identification have been carried out with intact and macroscopic specimen sample images or using the soft wing venation patterns. However, research on bettle species identification through their elytra fragments has not been undertaken especially for a systematic and integrated feature analysis. In this study, we aim to identify the food contaminating beetles at the species level through regconizing patterns in microscopic images of their elytra fragments.

## Data and Methods

**Fig 1:**
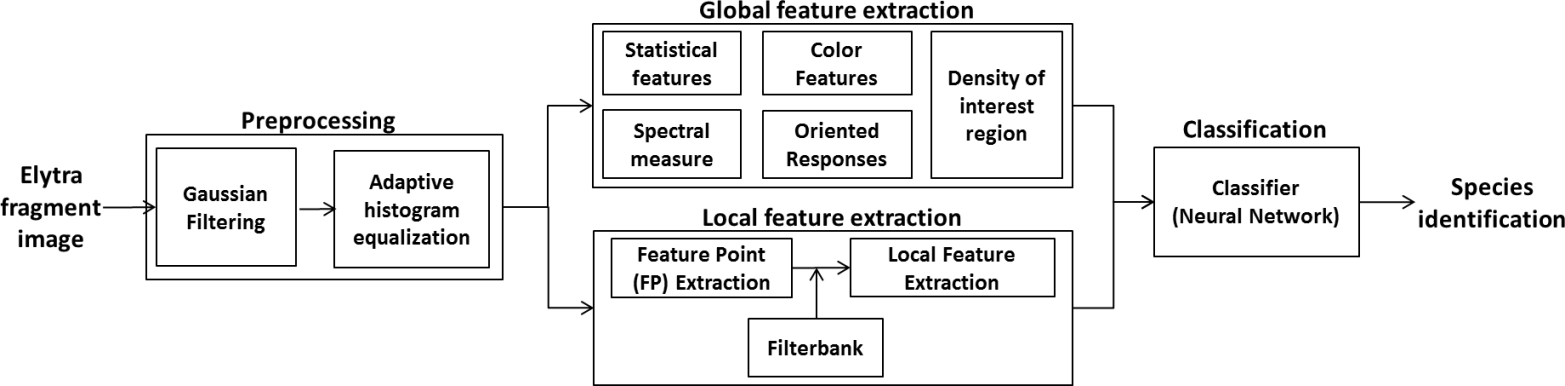
The workflow of food contaminating beetle species identification by image analysis.

Our algorithm for beetle species identification is outlined in Fig 1. As is typical for digital image processing and pattern recognition/classification, the envisioned workflow includes step-by-step procedures: preprocessing, feature extraction, and classification. As shown in Fig 1, the captured elytra fragment image is first Gaussian-filtered and histogram-equalized for quality enhancement. Then, global and local appearance characteristics are quantified as features, which are aggregated as a final feature vector. Finally, through normalization of all independent features, the (input) feature vector is fed into a neural network classifier for training. The rest of this section describes in detail data acquisition, observed elytra patterns (types), feature extraction, and classification.

### Data acquisition

As a preliminary effort, we chose 15 beetle species commonly found during food inspection and collected whole body specimens. The elytra were then taken off and imaged under a Leica M205 FA digital microscope (Leica Microsystems GmbH, Wetzlar, Germany); 3 to 6 elytra specimens per species were then chosen along with their images. Each specimen was manually positioned; then front and back images of each specimen were captured at magnification between 75X and 100X. In this study, only the front images were used. Table 1 lists 15 target species and the number of specimen images retained after discarding those of poor quality.

**Table 1:** 15 target species and the number of specimens collected.

Index (Number)	Scientific Name	Number of whole elytra images
1	Cryptolestes pusillus	5
2	Lasioderma serricorne	5
3	Gnathocerus cornutus	4
4	Zabrotes subfasciatus	6
5	Oryzaephilus mercator	4
6	Oryzaephilus surinamensis	4
7	Rhyzopertha dominica	4
8	Sitophilus granarius	5
9	Sitophilus oryzae	4
10	Stegobium paniceum	5
11	Tribolium breviconis	5
12	Tribolium castaneum	3
13	Tribolium confusum	5
14	Tribolium freeman	5
15	Tribolium madens	5

### Examination and categorization of elytra patterns

**Fig 2:**
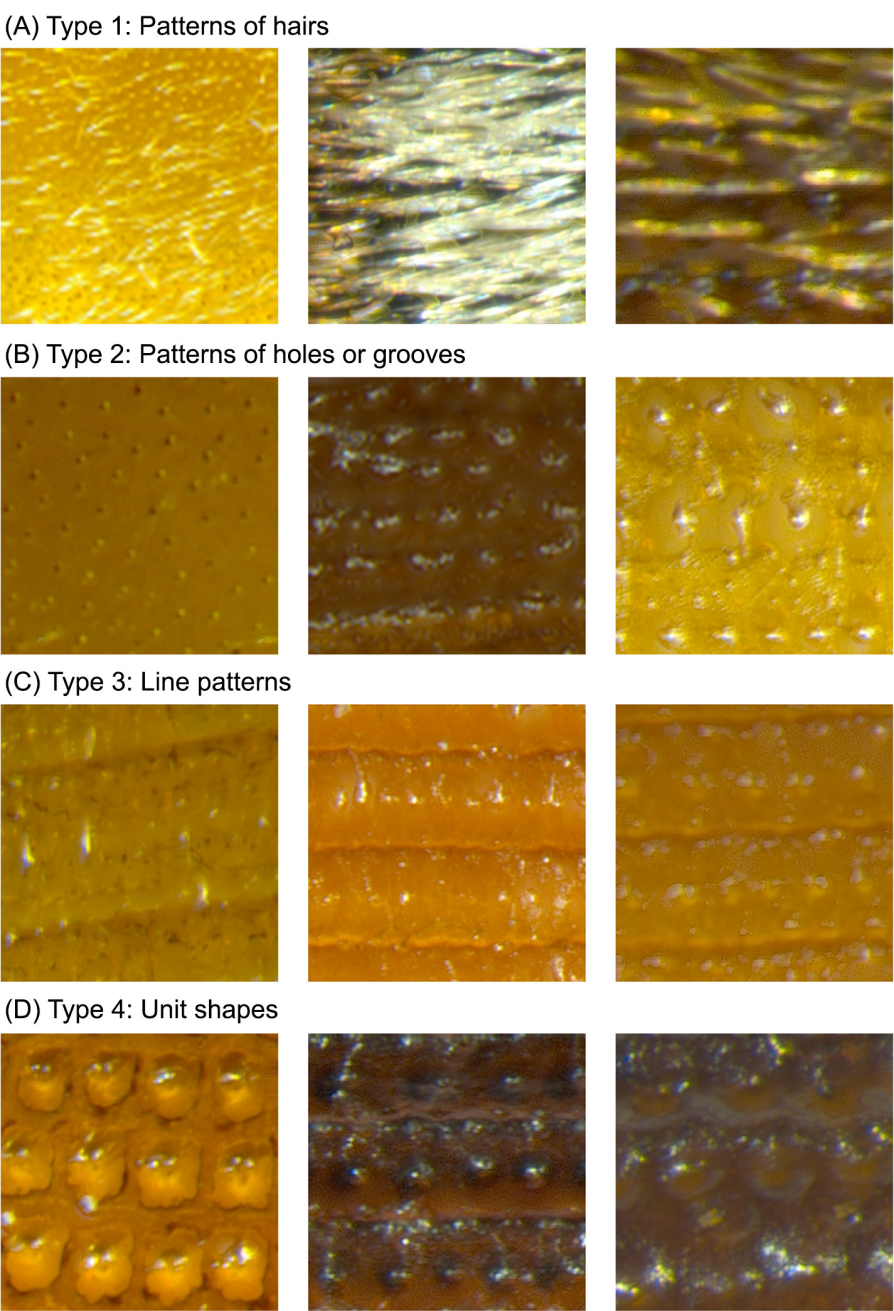
Categorization of elytra key characteristics.

To identify beetle species by recognizing patterns on elytra fragments, we first examined elytra key characteristics of the target species. In particular, we classified them into four different types or categories. The criteria for categorization are the existence of hairs, holes/grooves, lines, and unit shapes (e.g., circles, squares). Fig 2 shows the categories and some example images of elytra fragments.

Naturally, one species may exhibit characteristics from more than one category. Individual variations and partially damaged patterns due to food processing (e.g. grinding) may result in alteration to the characteristitic and possibly different type categorization. More importantly, some species that belong to the same genus possess similar visual characteristics, presenting a great challenge for species identification. For example, Species 5 (*Oryzaephilus mercator*) and 6 (*Oryzaephilus surinamensis*) belong to the same genus and have highly similar characteristics. Another example is the set of Species 12, 13, and 14 (*Tribolium castaneum*, *Tribolium confusum*, and *Tribolium freeman*). Since they exhibit extremely small visual differences across species, the established procedures of identifying beetle species based on hierarchical conditions/decisions or comparison of an elytra fragment image with reference elytra images may lead to inconsisten results. Fortunately, each species has its own combinations of elytra key characteristics with particular ranges of color or mathematical (numerical) quantities/relationships of the featured components which disambiguate similar appearance between species. Therefore, in addition to the types in Fig 2, we examined species-dependent characteristics and particular ranges of color domain values presented on hairs, holes, lines, and etc.

### Preprocessing and simulation of elytra fragments

Unlike the macroscopic images used in the prior research mentioned in Section 2, our elytra images are captured in a 3-D microscopic environment (i.e., depth-sensitive microscopy). Due to the curvature of the elytra, some local regions of the images are blurred or out of focus. In addition, different exposure, angle, intensity, or reflection of light when images are acquired can cause biased contrasts/intensities or saturated area. The curved area and the light effect lower the quality of images, causing blurred area, local peaks (salt noise), and varied contrasts. Thus, we preprocess the fragment images through a 2-D rotationally symmetric low-pass Gaussian filter (variance = 2, window size = 10x10) and adaptive histogram equalization to reduce local peaks and enhance the range of intensity, respectively. The preprocessing is aimed to remove noise and enhance contrast.

To segment an elytra region (foreground) from the background, two complementary binary masks are computed individually and merged. One mask is based on histogram analysis and the other is obtained from color similarity measurement as described below.

A histogram-based mask is created based on histogram peak-valley analysis. Usually, the intensity histogram of our collected images comprises two major (global) peaks; one peak indicates background-related intensities/pixels and the other indicates elytra-comprising (foreground) pixels. To facilitate finding global peaks in the histogram, a smoothing operation is needed to remove local peaks and valleys. We employ a 1-D Gaussian filter with a dynamic variance; the variance is calculated by averaging distances between adjacent local peaks. A valley of minimum value between the two global peaks on the smoothed histogram serves as a threshold to binarize the elytra region.

Based on color similarity measurements, a color-distance-based mask can also be created. Assuming the elytra object is located and captured in the middle of the image, we set a reference area (i.e. region of interest) in the middle (size: 2% of width x 2% of height) and get a reference vector from the RGB triplet histogram by averaging color component values with a fixed number of bins in the reference area. If a pixel is within a certain threshold (distance) when comparing the color component values of the pixel to the reference vector, the pixel is considered as an elytra pixel. In this manner, foreground pixels are selected for the mask.

Since there may be holes within the computed masks or background pixels falsely detected as foreground pixels in the elytra region, morphological operations, such as ‘filling’ and ‘opening’, are subsequently performed on each computed mask. Then, an ‘AND’ logical operation is applied on the two masks to obtain their complementary effect; this gives a more precise boundary between the elytra region and the background. After filtering the whole elytra image through the final binary mask, the elytra region is segmented. Within the detected elytra region, subimages with random sizes (i.e. random width and height) from random positions are repeatedly selected to simulate various elytra fragments; we preset minimum (= 300 pixels) and maximum (= 2500 pixels) width and height for the random size. In our simulation, we take 100 subimages per elytra specimen image in order to cover a whole elytra area in a sufficiently balanced way.

### Global feature extraction

Detecting and recognizing patterns of the elytra fragments requires two stages of processing: the feature extraction stage which uses digital image processing, and the classification stage. Whole beetle elytra or the fragments include globally appearing characteristics/features such as color, and strong or directional edge, etc. In order to obtain the global features, various methods, such as Fourier transform, Difference-of-Gaussian, and Gabor filter are employed. Overall, these global characteristics are computed with mathematical metrics and defined functions.

As mentioned in the species identification procedures [19], the global feature approach is to describe a global appearance which contains the overall characteristics of a whole elytra fragment. To quantify the global appearance, several metrics are computed: statistical and spectra measures, color distribution, oriented edge response, and density of specific objects of interest such as hairs, holes, and lines. The computation of the individual features is addressed in more detail in the next subsections.

#### Statistical (Spatial) features

To describe the global appearance, we employ several statistical measures that represent discriminant texture properties using a 1-D intensity histogram and 2-D moments. Such measures of elytra fragment images are based on texture analysis in the spatial domain. Based on previous research [20, 21], the following measures are collectively employed as one of the global appearance descriptors. Except Hu moment [22], each of the statistical features is computed as one dimensional numerical output value.

- Mean: a measure of average intensity
- Standard deviation: a measure of contrast
- Uniformity: a measure of uniform grayness (maximum value when all gray values are equal)
- Entropy: a measure of disorder/randomness
- Smoothness: a measure of relative smoothness of the intensity
- Third moment: a measure of the skewness of the histogram
- Hu moment: 7 moment invariants with respect to translation, scale, and rotation.

#### Spectra features

**Fig 3:**
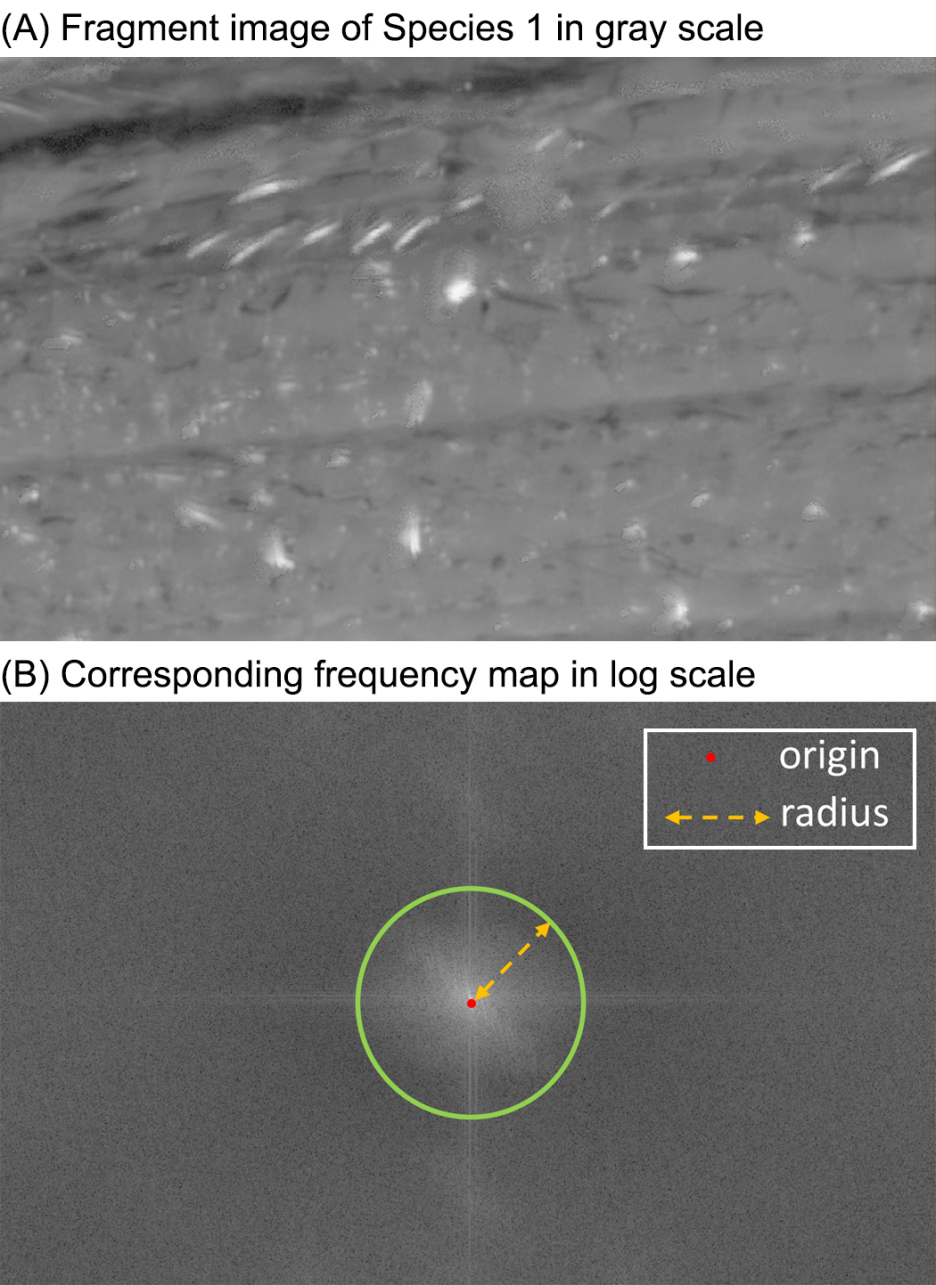
An example of elytra image in gray scale and the corresponding frequency map.

With the spatial texture measures, it is difficult to discriminate whether elytra unit patterns are periodic or non-periodic. A better approach is to measure spectra properties in terms of pattern periodicity by transforming spatial domain to frequency domain; this takes into account the directionality of periodic patterns and the concentrations of low or high frequency/energy in the spectrum. Using two dimensional discrete Fast Fourier Transform [23], we calculate frequency responses. To describe the spectra features in two-dimensional frequency domain, we sum up absolute frequency responses (i.e. Euclidean norm of real and imaginary part coefficients) for different radii from the origin of the transformed frequency map. As shown in Fig 3, for each radius *r*, the corresponding spectra responses in feature map are computed by Eq 1.

$$Spectra\_Feature(radius=r)=\sum_{\theta =0}^{2\pi }\left\|FM(r,\theta )\right\|,$$ (1)

where *FM* stands for two dimensional frequenecy map and *(r*, *θ)* is the coordinate in polar with radius *r* and angle *θ*.

Since we exclude the component of zero frequency (i.e., the dc component) and dominant frequency patterns are observed within radius of 80, we set radius *r* from 1 to 80. The results of these measures serve as another global feature set.

#### Color features

**Fig 4:**
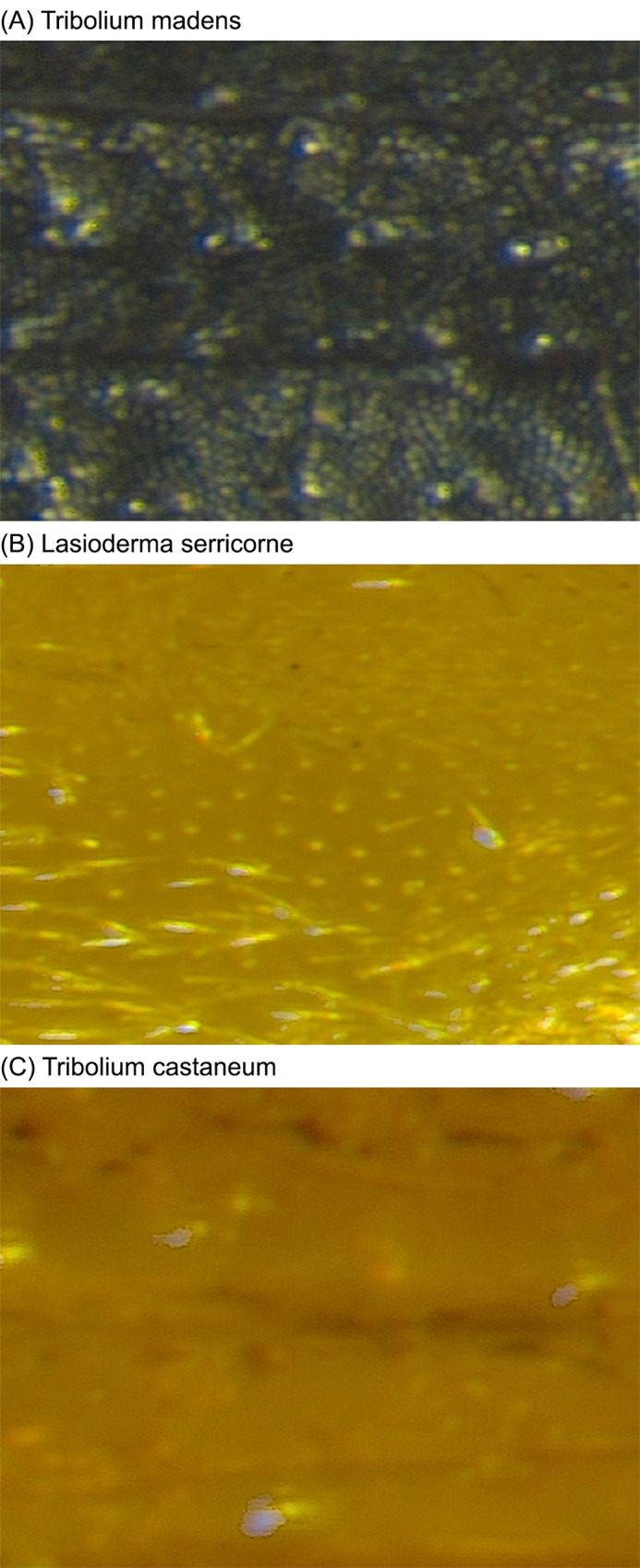
Examples of elytra colors.

Along with the texture analysis in spatial and spectrum domain, color distribution of elytra is invariant or relatively insensitive to surface orientation and illumination. (Note that the elytra images are collected in a lab environment.) Color is one of the key elements used to distinguish between species. Fig 4 shows examples of different elytra colors. As shown in Fig 4, elytra of *Tribolium madens* (Species 15) have a different color from those of species *Lasioderma serricorne* (Species 2) or *Tribolium castaneum* (Species 12).

We digitized RGB color components in RGB domain and concatenated the histogram in each domain as feature vector elements [24]. The number of bins in each color domain is set to 10 not only to represent various elytra colors of 15 species, but also to cover a range of elytra color variation in each species.

#### Oriented responses using Gabor filters

Due to the radial or linear directional arrangement of edges in elytra compartments, another texture representation of elytra patterns involves oriented (angular) impulse responses. Gabor filters quantitatively characterize edge structures with orientations. An oriented Gabor filter *G*_*θ*_ and the oriented responses *C*_*θ*_ are formally defined in Eq 2.

$$G_{\theta }(x,y)=exp\left(-\frac{{x\prime }^{2}+\gamma^{2}{y\prime }^{2}}{2\sigma^{2}}\right)exp\left(i(2\pi \frac{x\prime }{\lambda }+\psi )\right),$$

$$C_{\theta }(x,y)=G_{\theta }(x,y)*I(x,y),$$ (2)

where *θ* is the orientation of the Gaussian envelope; *x* and *y* are the spatial coordiantes; *x′ = xcosθ+ysinθ* and *y′ = -xsinθ+ycosθ*; *λ* is the sinusoidal factor; *ψ* is the phase offset; *σ* is the sigma/variance of the Gaussian envelope; “*” represents the convolution operator; and *I(x*,*y)* stands for the pixel intensity at *(x*,*y)*.

**Fig 5:**
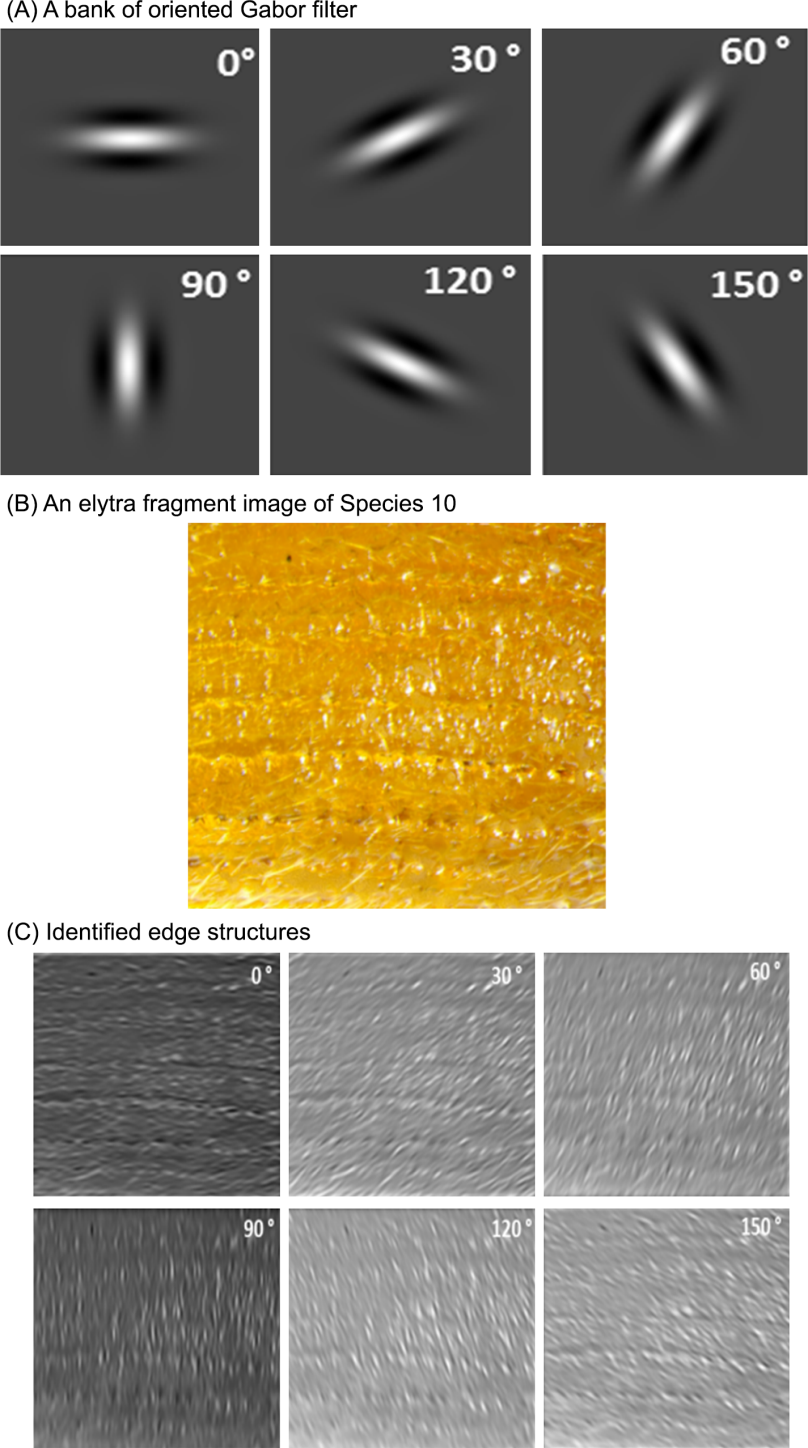
An example of angular responses using oriented Gabor filters.

We apply Gabor filters to the elytra fragment images with preset orientations [25]. The image is convolved with a bank of oriented Gabor filters G_θ_ with orientation θ, where 0° < = θ < 180° in increments of 30°. Fig 5 shows a bank of Gabor filters and the corresponding filtered results after applying the filters to a fragment image (Species 10 in Table 1). As shown in Fig 5, after using the oriented filters, edge structure is identified. We present the prominent edge structure with the sum, mean and standard deviation of the convolved images. The sum, mean, and standard deviation values are reflected on the feature vector. Additionally, the corresponding angle with the maximal response is added to the feature vector. Thus, the Gabor descriptor consists of 19 elements, the sum, mean and stanadard deviation for each of six oriented Gabor filters, and the max response angle.

#### Hair/Hole/Line features

As mentioned above, hairs, holes, grooves, or line patterns are a distinctive class of elytra features. These components are perceived visually since they stand in contrast to the surroundings. To segment these feature objects, we employ a Difference-of-Gaussian (DoG) filter with two variances: one large and one small, i.e., 100 and 1, respectively. A Difference-of-Gaussian filter is defined as follows:

$$DoG(x,y)=\frac{1}{2\pi {\sigma_{1}}^{2}}e^{\frac{-(x^{2}+y^{2})}{2{\sigma_{1}}^{2}}}-\frac{1}{2\pi {\sigma_{2}}^{2}}e^{\frac{-(x^{2}+y^{2})}{2{\sigma_{2}}^{2}}},$$ (3)

where *x* and *y* are the spatial coordiantes; *σ*_*1*_ and *σ*_*2*_ are the two variances for the Gaussian envelopes.

**Fig 6:**
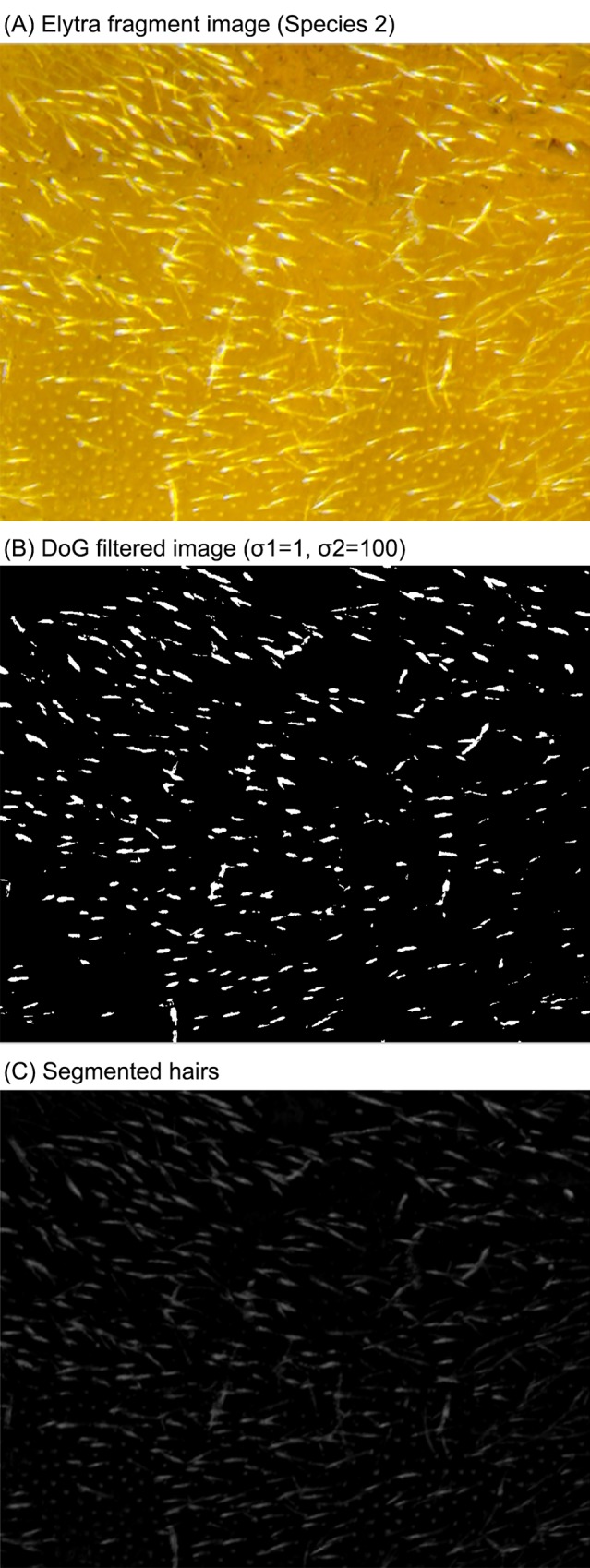
An example of elytra hair like feature detection.

The equation implies that a largely blurred image is subtracted from a less blurred one and vice versa, thus, two DoG filtered images are used. Through the DoG filter, we approximate higher and lower intensity of the objects/regions. Then, the approximated objects (i.e. regions of feature elements such as hairs and holes) are detected [26] and binarized; a dynamic threshold for binarizing the DoG filtered image is calculated by averaging Otsu thresholds [27] computed from a gray-scale original image and the DoG filtered image. Fig 6 shows a sample fragment image (Species 2), the DoG filtered image, and the binary segmented image. Finally, from the binary image, color distribution, number and area density, and average and median size of the objects are computed. Specifically, color distribution is represented with 30 bins (10 bins in each domain) in RGB color histogram only for segmented hair/hole/line objects. Number and area density are obtained by dividing the total number and area of the segmented objects by the total submiage area, respectively. The final feature vector include 68 elements: 30 dimensional color disciptor, number & area density, mean & median for each of the two DoG filtered images. Notably, along with color distribution, the density features identify and quantify the amount of local key objects that are not attentively measured in spatial/spectra texture or color descriptors. Since they are computed throughout the whole captured area, but takes in only local key regions, we categorize this feature set into another type of global feature as “global feature 2” in Results section, which lead us to explore the impact of key objects.

### Local feature extraction

The global features are directly calculated from the whole elytra fragment images. However, these features may not accurately represent local unit patterns (e.g. rectangle, circle, and hole). Also, such features may be affected by factors such as captured/simulated area and/or the size and rotation of the simulated fragment image. In order to express the local appearance precisely, certain important points such as centroids of holes, hairs and unit shapes are selected; then a confined local area around the points of interest is processed for the local appearance analysis. We call such selected points feature points and restrict a process within the specific local area around each feature point using a filter bank. In addition, to represent repeating unit patterns or unique hair/hole/line patterns found locally, mask (window) operations around the feature points are employed. Specifically, a corner detection method for feature points and various computations (e.g. vertical/horizontal projection) around the feature points are performed.The next two sections address the feature point detection and filter-bank-based local feature extraction.

#### Feature point detection

As the first step to describing local structures, we need to define interest points, i.e., feature points that can be detected in consistent and reliable positions. In particular, the points should be tolerant of scale variation to some degree, as it can often happen in microscopic images. In addition, rich local structural content around the feature points needs to exist for its topographical significance. Considering these requirements, we employ the concept of corner points. Among several mathematical approaches, we chose to use Harris corner detection [28] to define and detect corner points. A corner point is defined as the intersection of two edges; shifting a window in any direction from the corner yields significant changes. Thus, sufficient local structure features around the corner can be obtained.

**Fig 7:**
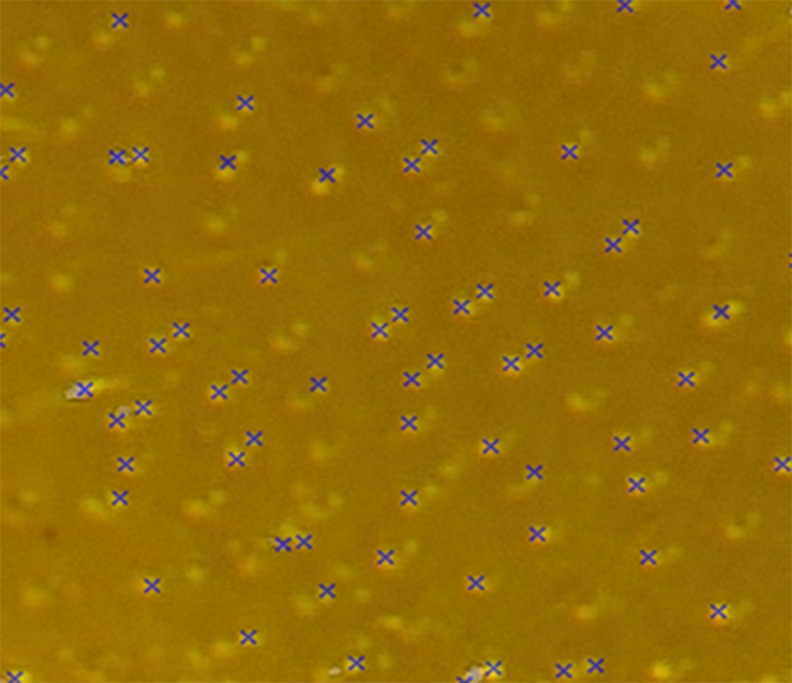
Example of detected feature points (marked as blue ‘X’s).

In practice, a large number of the feature points (over 300 feature points per image) can be initially detected in the fragment images. Many adjacent points within a certain distance from each other (15 pixels) may need to be merged. To refine and merge the feature points, feature points of high density are rejected since undesired false corner points are observed densely in local peak-noise area. For this rejection, we calculate the average number of corner points per window area and set up a threshold range based on the average, i.e., 25% truncated mean calculation. The feature points are accepted only within an area within the range. Also, to merge adjacent points, the individual feature points are dilated with a disk structured element (radius = 4 pixels) and the connected components are subsequently analyzed. Centroids of the individual connected components are located as final feature points. Fig 7 shows an example of detected feature points on holes in a Species 2 fragment image.

#### Local feature extraction using filterbank

**Fig 8:**
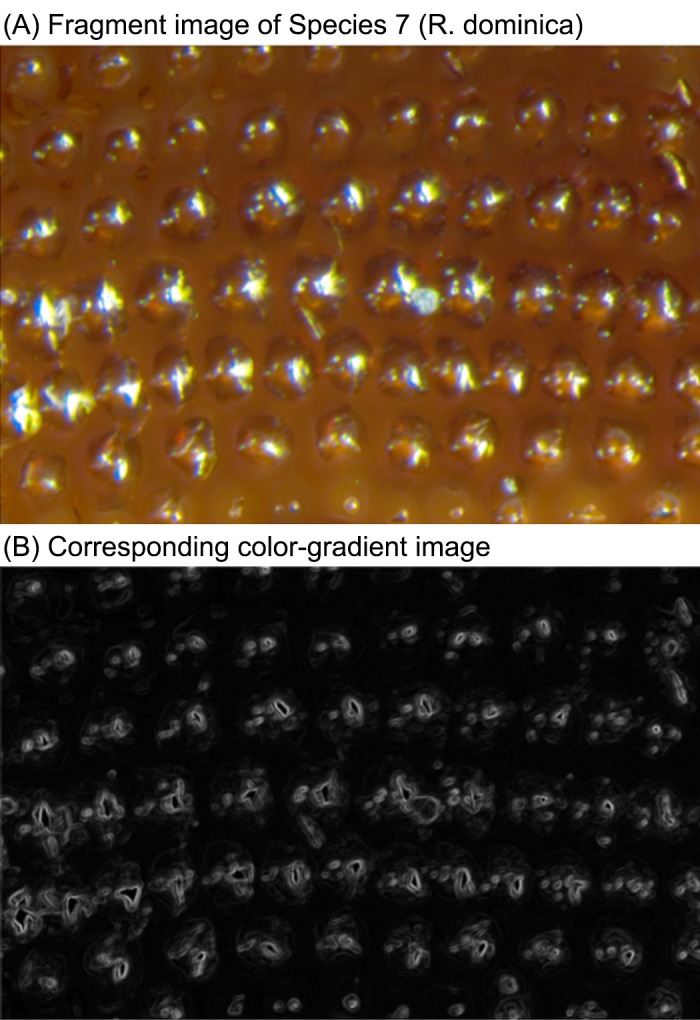
Sample elytra fragment image of Species 7 and the corresponding color-gradient image.

To represent local structures around the feature points, color gradient-based edges [27] are computed. Fig 8 shows an elytra fragment image (Species 7) and the color-gradient-edged image.

**Fig 9:**
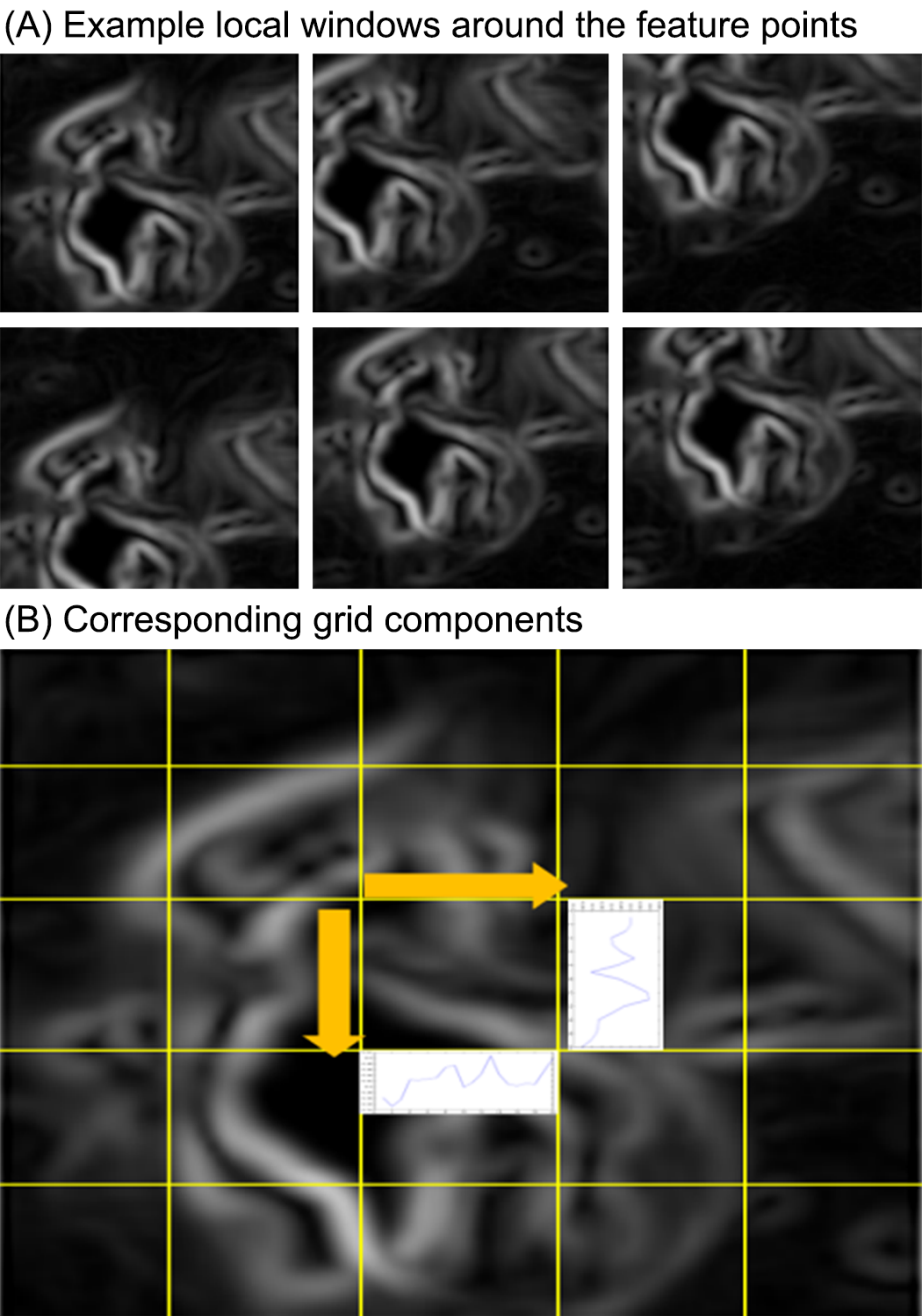
Example local windows around the feature points and the grid components.

From the edge image, a window of preset size (101 x 101 pixels) centered at each feature point is constructed. The window (i.e. local area) is then divided into multiple grid components; Fig 9 shows example captures of the local windows and the individual grid components.

**Fig 10:**
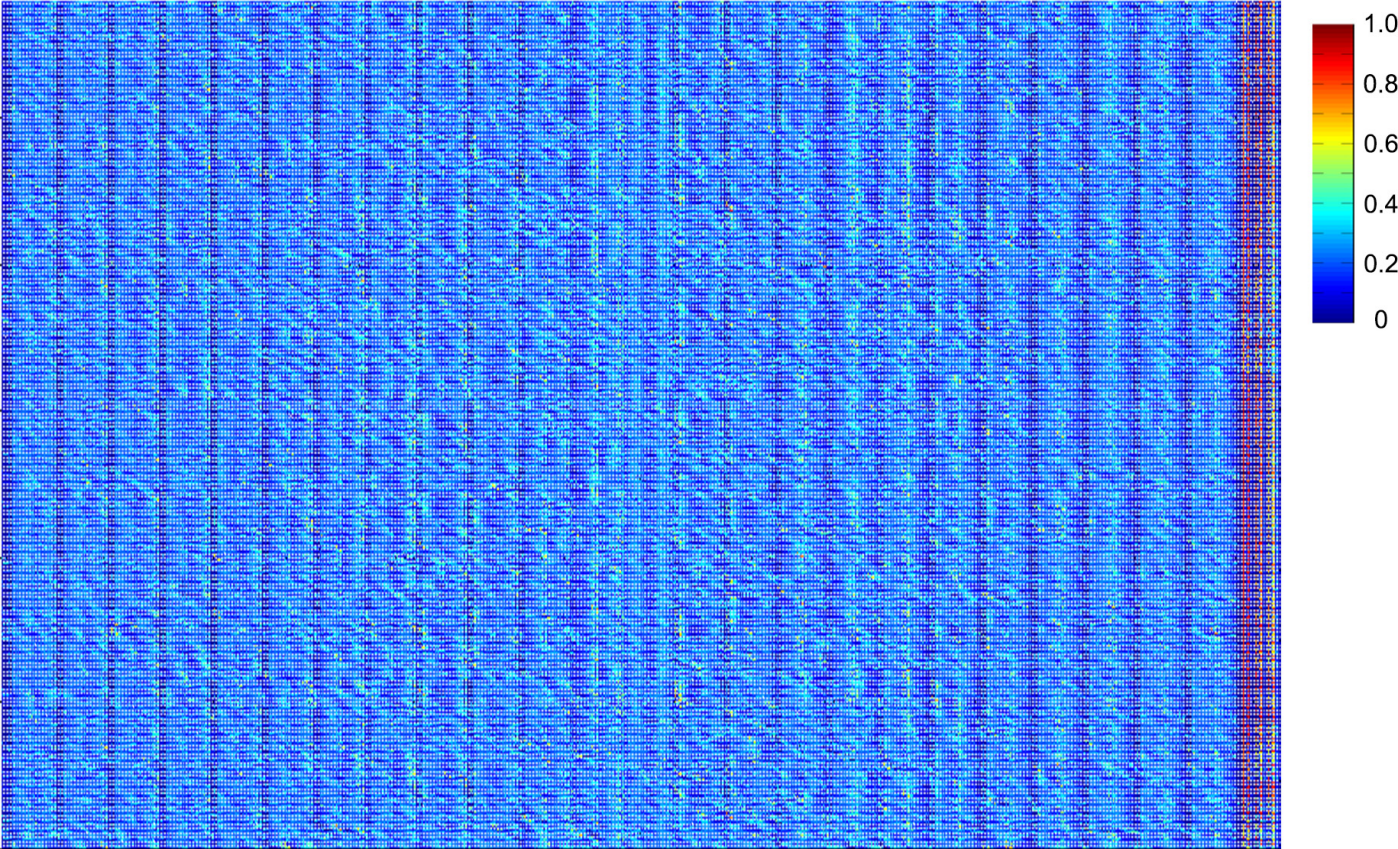
An example heatmap of final computed local feature vectors.

After division, an array of filters and processing (i.e., filter bank) is applied to each cell. The operations within each partitioned area (20x20-pixel grid for each partition) include (1) horizontal and vertical histogram projections with interpolation (7 data points for each x,y axis), (2) mean and variance calculation, and within the individual local windows, color distribution in RGB domain (number of bins is 5 in each domain, total 15 bins). The resulted values are all concatenated in a vector. Finally, the computed vectors from individual local areas (i.e., from each interest point) are averaged for a final local feature set. The individual local feature vectors are illustrated in Fig 10; the y-axis and x-axis present the individual local windows centered at feature points and the corresponding local feature vectors, respectively. As shown in Fig 10, a discernible pattern in the local feature vectors is observed along the y-axis.

### Species classification using artificial neural networks

To recognize beetle species, after extracting and normalizing the global and local feature sets, we employ a feed-forward neural network classifier with two hidden layers in sigmoid activation function. Both feature extraction and classifier development were implemented through MATLAB v2012a (MathWorks, Natick, Massachusetts, USA). The global feature vector is composed of the following features with the number of elements shown in parentheses: statistical (spatial texture) features (13), spectra features (80), color features (30), Gabor features (19), and hair/hole/line density feature (68). In total, the global feature vector has 210 elements. As for the local feature vector, 16 elements from each of 25 (5x5 grid) partitions are used to represent mean/variance (2), vertical/horizontal projection interpolated (14: 7 for x and y, respectively). In addition, 15 color bins in the local window (5 bins for each color domain) are extracted. The local feature vector has 415 elements. The feature extraction for each fragment image took 1 to 3 minutes, depending on the image size, using multithreaded and multiprocessor programing on a workstation with two Intel Xeon 2.3GHz 6-core CPUs and 128 gigabytes of memory. To assess the effects of the global and local features on performance, or classification accuracy, we grouped the features into four sets: (1) a subset of global features except hair/hole/line features, (2) the complementary subset of global features (i.e., hair/hole/line features), (3) the set of local features, and (4) the combined set of global and local features (all computed features). In particular, to observe how effectively the features obtained from selectively-detected objects such as hairs and holes represent the elytra characteristics, the first subset of global features does not include the hair/hole/line density feature.

The data comprise 6,900 elytra fragment images (100 fragment images per whole elytra image from the collected specimens, total about 30 gigabytes). For each round of cross validation, one specimen in each species is randomly and independently reserved to make the hold-out test set. Thus the numbers of training and test images are 5,400 and 1,500, respectively. We first explored a few ANNs with one, two, or three hidden layers for 10 rounds of cross validation and found that networks with two hidden layers usually delivered better classification performance. Following the rule of thumb method [29], we experimented with the number of nodes in hidden layers in multiples of 25, ranging from one fourth to two times of the dimension of each feature set. Our exploration led to the configuration choice of two hidden layers with 50 nodes at each layer. To improve neural network generalization and avoid overfitting, we adopted the early stopping technique implemented in MATALB with random division of 5,400 training subimages by 70:20:10 into three sets to monitor the training progress. This early stopping approach, together with the relatively small network configuration, also greatly reduced the training time to under 15 minutes. With each feature set, we trained one ANN and tested it by the hold-out test images. This was then repeated 100 times during cross validation to gauge the performance achieved by each feature set and how it may vary per species.

At the end, we explored two commonly used methods to select informative features and compared the performance of selected feature subsets with the whole set. The minimum redundancy maximum relevance (mRMR) method [30] selects a feature subset based on the combined relevance and redundancy that are defined by high-dimensional mutual information. It offers two options for residual relevance quantification to rank the selected features: mutual information difference (MID) or quotient (MIQ). We selected 50 features using each option and tested the cross-validation identification accuracy using the top K features with an increment by 5. The second method, correlation-based feature selection [31], selects a subset of features by considering their individual predictive ability and the degree of redundancy between them. It has a forward and backward option to output a feature subset without ranking. We acquired two feature subsets through the correlation based method. All feature subsets were then tested with the same 100 rounds of cross validation to compute identification accuracy for comparison with the results achieved without feature selection.

## Results

**Fig 11:**
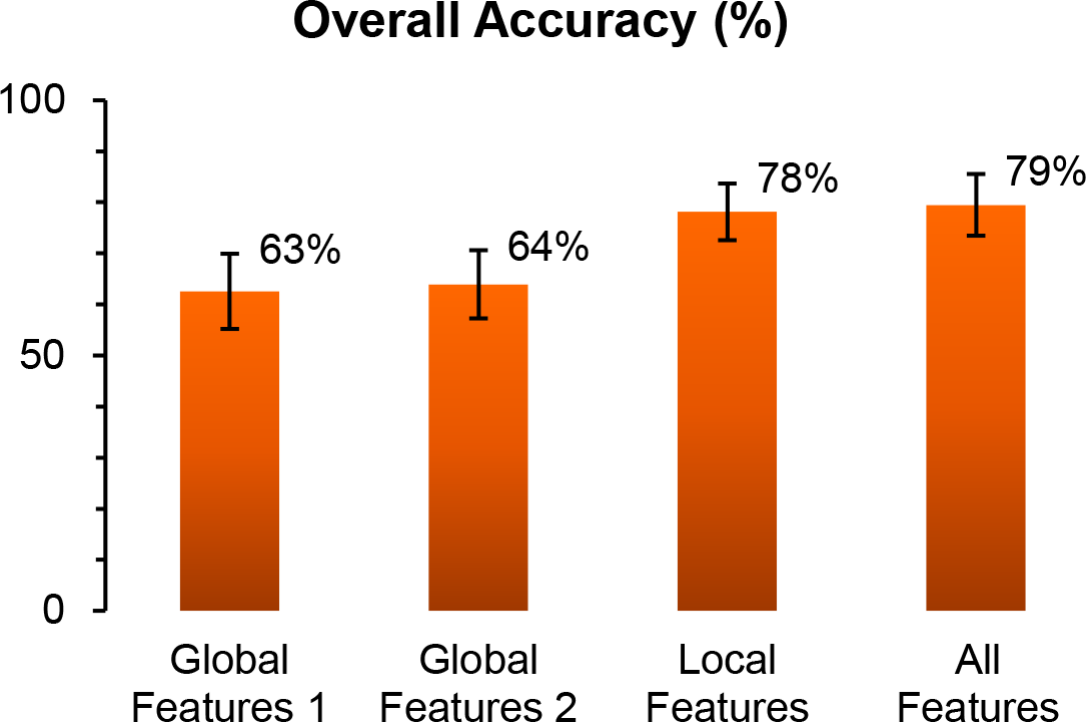
Overall accuracies of species identification using four different feature sets. Each bar plots the mean and the standard deviation of the overall accuracy achieved during 100 rounds of cross validation.

Fig 11 shows the overall identification accuracy for each of four feature sets, which are labeled “global features 1”, “global features 2”, “local features”, and “all features”, respectively. Networks built with all features achieved the highest overall identification accuracy (with a mean accuracy at 80%). When comparing the results between “global features 1/2” and “local features”, and those between “local features” and “all features”, the global features performed worse than the local features and seemed to contribute little to the overall performance achieved by “all features”. Furthermore, except two or three rounds of cross validation, local features consistenly outperformed the global features 1 and 2 (data not shown). This result implies that the local features are more likely than the global features to capture the elytra characteristics specific to each species. In addition, it supports the interpretation that the global features might be more sensitive to overall data quality than the local features computed around the refined and merged feature points. In turn, the necessity of collecting image data with consistent quality needs to be emphasized for further research.

**Fig 12:**
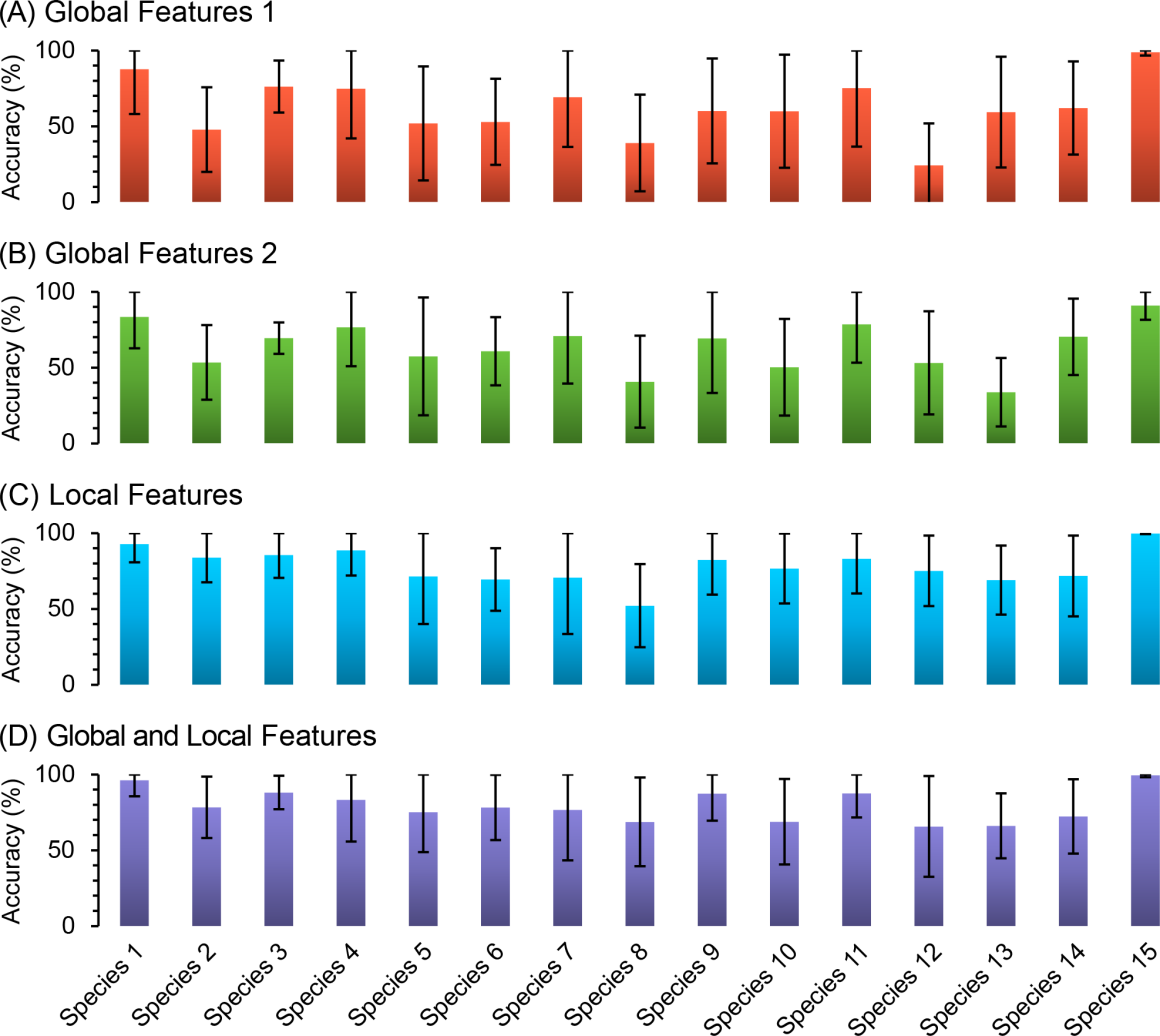
Identification accuracy per species. One panel plots the results for each feature set: (A) a subset of global features (i.e., “global features 1”), (B) the second subset of global features with hair/hole/line features (i.e., “global features 2”), (C) the set of local features (i.e., “local features”), (D) all features (i.e., “global and local features”). Each bar plots the mean and standard deviation of identification accuracy achieved during 100 rounds of cross validation.

Along with the overall performance, we examined identification accuracies for each species. Each subpanel of Fig 12 shows per species accuracies obtained by the global features 1 or 2, local features, or all features. The statistical, spectral, color, and angular edge response features in the “global features 1” were capable of identifying many species, but they were not sufficient to distinguish species with a high resemblance. Even with hair/hole/line quantificaiton, the “global featuers 2” rendered a meager performance improvement. The better performance of the local features demonstrated that the segmented local attributes represented in the local features were most effective for characterizing individual species that look alike. Besides a small improvement in overall performance, the inclusion of the global features led to more balanced per species performance than the local features alone. The lowest per species mean accuracy was 66% (for Species 12) by all features but only 52% (for Species 8) by the local features. With all features, 5 species (Species 1, 3, 9, 11, and 15) were identified with high accuracy (≥ 85%) and 5 species (Species 2, 4, 5, 6, and 7) were well-classified (75%–85%). Species 8, 10, 12, 13, and 14 were recognized with decent accuracy (65%–75%). Importantly, Species 12, 13, and 14 were classified correctly at genus level due to their very similar characteristics as mentioned earlier. Thus, we confirmed that some objects of interest such as hairs, holes and lines were crucial features for species identification.

**Fig 13:**
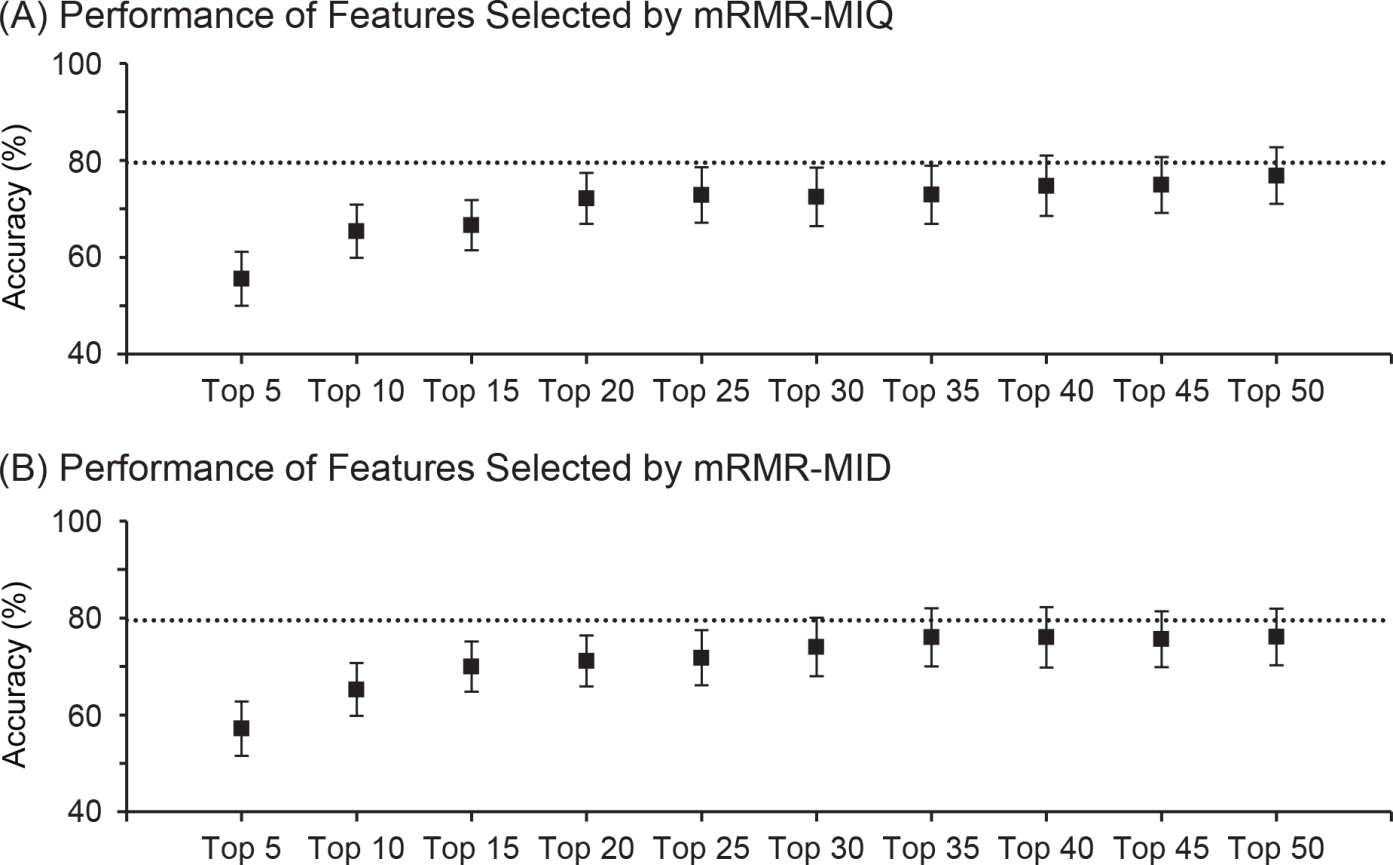
Cross validation identification accuracy of the features selected by mRMR. Each panel plots the results for: (A) the top K features selected through the option of MIQ, and (B) the top K features selected through the option of MID. Tests were done for K’s in multiplies of 5. The dot and error bar plot the mean and standard deviation of identification accuracy achieved during 100 rounds of cross validation. As a comparison, the dotted lines represent the mean identification accuracy achieved by all features without feature selection.

Feature selection was conducted among all global and local features. The cross validation identification accuracy was plot in Fig 13 for feature subsets selected by mRMR. Since the features were ranked, we were able to test their performance incremently as more features were included for testing. For the MID scheme, there was almost no performance gain beyond the top 35 features while the gain was marginal for the MIQ scheme. The best performance, 76.9% for MIQ or 76.1% for MID, was achieved with all 50 selected features. However, both of them were still below the mean accuracy (79.5%) achieved by all features without feature selection, which is plotted as dotted lines in both sub figures. Furthermore, different network configurations such as one or two hidden layers with 25, 50, or 75 nodes were also tested for all 50 selected features. Yet no performance improvements were observed (data not shown). As mentioned above, the correlation-based feature selection method has forward and backward options when each feature is evaluated for inclusion or removal. The forward option selected 52 features while the backward option output 55 features. Since no ranking information was given for these features, we only tested each selected feature subset through the same 100 rounds of cross validation. The mean and standare deviation of identification accuracy were 72.8% and 5.5% for the forward subset, or 69.1% and 5.3% for the backward subset. Thus these two commonly used feature selection methods provided no benefits in improving the classification accuracy for our data set. mRMR appeared to work better than the correlation-based method.

In summary, our results demonstrated the effectiveness of using global and local features for beetle species identification, as evidenced by the high accuracy rates. In addition, the local features led to a considerable performance gain and thus were more effective than the global features.

## Discussion and Future Work

We started this work with the goal of developing a software algorithm for identifying beetles from their fragments at species level; the aim of this project was to support food inspection. To this end, we selected 15 common storage beetle species and acquired microscopic images of their whole elytra specimens. We then categorized the elytra keys and explored synthetic features to model them. Finally, using ANN classifiers, we evaluated the extracted features in light of their effectiveness in beetle species identification. Our results sufficiently demonstrated the feasibility of applying image analysis to further beetle identification research.

As the elytra (or fragment) images were captured by a microscope, there are several challenges we discovered. First, we need to collect better quality data. In order to standardize the quality, a formal process for acquiring image data (i.e., data acquisition protocol) needs to be established. In addition, we need to preserve the actual fragments obtained during food inspection and build a database to store the various images.

With regards to the elytra keys and computed features, we examined and quantified the elytra characteristics, then explored the effect of various features correspondingly. We evaluated the effectiveness of the global and local features individually and in combination. The global features were effective at identifying species not of the same genus, or that have distinctive shapes, textures, and colors across a whole elytra fragment. On the other hand, due to the weakness in describing a subtle local appearance with global features, the local features were useful to differentiate between similar species that have similar hairs/holes/lines, or are of the same genus. In addition to analysis of the current features, we intend to find more candidate features that can be used for identification to further improve the accuracy for more challenging species such as Species 8 and 10. For example, the average distances from each feature point to the most adjacent K feature points or their density (i.e. distribution of corner points) can serve as new features. With the release of image data along with this publication, we hope to stir up interests in this important research field that has a tremendous impact on safeguarding our food supplies.

According to our small experiment with different configurations, ANN showed fast and fault-tolerant classification performance. With the early stopping technique implemented in MATLAB, the learning process in ANN usually took no more than an hour even for a large ANN (using the same workstation with two Intel Xeon 2.3GHz 6-core CPUs and 128 gigabytes of memory). Given the result that the performance is more dependent on feature sets and species, our future effort will be focused on data expansion and exploring additional local features. After the planned data expansion that is discussed below in detail, we will explore various approaches to optimize the ANNs and perform extensive testing. One drawback of the current ANN design is that any test image will be classified as one of the 15 species included in the study data collection. Though data expansion will help elleviate this shortcoming, we may explore more ANN design options or other classification methods to better address this issue. Some classification methods, e.g., SVM and convolutional neural network [32], will be tested and compared with ANN. For example, SVM was recommended for real application deployment due to its better performance over ANN while ANN would be more useful for testing new features [13].

It is important to note that our current study has some limitations. First, there are 3–6 specimen images per species. A significant increase of specimen images will include more variation in specimen and thus afford a more robust test. Although the selected 15 species can identify the majority (about 70%) of storage beetle fragements recovered during food inspection, to enhance the regulatory utilities of this work, we plan to expand our specimen collection to include about 10 more species so that most (about 90%) storage beetle fragments could be accounted for. Finally, no beetle species from agriculture farms or gardens were included. Since agriculture species pose little risk to public health, we plan to select several agriculture species with elytra sizes similar to those of common storage beetles and incorporate them as negative controls. We expect these planned expansions in data collection will bring new challenges to feature discovery and classifier development.

In conclusion, our approach and efforts have yielded promising results of beetle species identification and demonstrated the feasibility to address the challenging species identification problem in food inspection with modern technologies. As our project and research continues beyond algorithm development, our focus will extend to building a beetle species identification system and a complementary elytra image retrieval system that has the potential to be useful for many different inspection and regulatory activities. We envision such a full-fledged system using state-of-art technologies: a distributed database (e.g., ElasticSearch [33]) for scalable and flexible data warehouse, GPU-based computation in the backend for fast computation [34], and an integrated user interface (e.g., [35]) in the front-end.
