# Correction: Species Identification of Food Contaminating Beetles by Recognizing Patterns in Microscopic Images of Elytra Fragments

**DOI:** 10.1371/journal.pone.0162757

**Published:** 2016-09-07

**Authors:** Su Inn Park, Halil Bisgin, Hongjian Ding, Howard G. Semey, Darryl A. Langley, Weida Tong, Joshua Xu

There is an error in the current address for author Halil Bisgin. The current address should be: Department of Computer Science, Engineering and Physics, University of Michigan-Flint, Flint, Michigan, United States of America.
